# Molecular Detection and Glycoprotein B (UL55) Genotyping of Cytomegalovirus among Sudanese Renal Transplant Recipients

**DOI:** 10.1155/2022/5403694

**Published:** 2022-05-31

**Authors:** Hind Haidar Ahmed, Hisham N. Altyab, Samar M. Saeed, Wafaa Mohammed Abdalla, Alfadil Elobeid Omer

**Affiliations:** ^1^Department of Microbiology, College of Medical Laboratory Science, Sudan University of Science and Technology, Sudan; ^2^Biochemistry Department, Faculty of Sciences, King Abdulaziz University, Jeddah 21452, Saudi Arabia; ^3^Department of Microbiology, Faculty of Medical Laboratory Science, Al Neelain University, Sudan

## Abstract

**Background:**

Cytomegalovirus (CMV) is the most common opportunistic pathogen among renal transplants with significant morbidity and mortality. This study was designed to detect CMV DNA and to determine the frequency of different glycoprotein B (UL55) genotypes among Sudanese renal transplant recipients.

**Methods:**

One hundred and four renal transplant recipients were included in this study. A blood specimen was collected from each recipient. DNA was extracted from plasma using the QIAamp DNA mini kit. CMV amplification and quantification were performed using CMV Real-RT Quant kits. Genotyping of human CMV *gB* was carried out by nested PCR and sequencing of the highly diverse region of *gB*.

**Results:**

CMV DNA was detected in 40/104 (38.5%) of renal transplant recipients. The average of the CMV DNA viral load was 358 × 10^4^ copies/ml (6.5 log_10_) ranging from 62 copies/ml (1.8 log_10_) to 1.43 × 10^8^ copies/ml (9 log_10_). CMV viremia was detected in 60% of recipients of less than 1–12 months, 17% of 13–24, 10% of 25–36, 5% of 37–48, and 8% in more than 48 months posttransplantation with no association (*p* = 0.296) between CMV viremia and postrenal transplantation time. The association between the type of immunosuppressive drugs and high viral loads (>1000 copies/ml) showed a significant difference (*p* = 0.05). The association between CMV loads of >1000 copies/ml and symptoms of CMV disease was highly significant (*p* ≤ 0.001). Fever 7 (41%), fever and leucopenia 6 (35%), and gastrointestinal disease 4 (24%) were the most common symptoms of CMV disease. CMV genotyping revealed 8 cases (80%) for *gB*3 and 2 cases (20%) for *gB*4 genotypes. The most frequent genotype among Sudanese renal transplant recipients was *gB*3.

**Conclusions:**

The frequency of CMV DNA is high among Sudanese renal transplant recipients. CMV *gB*3 is the most predominant glycoprotein B genotype in Sudanese renal transplant recipients.

## 1. Introduction

Human cytomegalovirus (CMV) is a ubiquitous double-stranded DNA belonging to the Herpesviridae family that establishes lifelong latency after primary infection and causes life-threatening disease in immunosuppressed patients [1]. Following renal transplantation, CMV has the greatest opportunistic viral morbidity other than any pathogen [2]. CMV replication in the graft after reactivation in the transplanted kidney or infection from the host [3] is a leading cause for allograft failure and mortality. Without prophylactic measures, 40%–100% of renal transplant recipients undergo CMV infection and about 67% develop CMV disease [2]. The effects of CMV infection on transplant recipients are classified to direct and indirect [4], which has been associated with active viral replication [5]. The major symptoms of direct effects include fever, neutropenia syndrome, and end-organ diseases such as pneumonia, enteritis, meningitis, and encephalitis. Cytokines, chemokines, and growth factors are released in response to viral infection of the body [6], which are immunomodulatory that intensify immunosuppression and increase the risk of other opportunistic infections considered as indirect effects [7]. An elevated level of viral load and viral load peak was associated with CMV disease in renal and liver transplant recipients [8]. Glycoprotein B is encoded by the CMV UL55 gene involved in several essential steps in CMV virus pathogenesis including virus cell penetration, cell-to-cell spreading, and activation of the immune response [9]. It has a main role in the activation of innate immunity as the major antigen for the induction of neutralizing antibodies. *gB* antibodies have been of interest because of their therapeutic potential for neutralization [10]. Regarding UL55 polymorphism, CMV has been allocated into 4 genotypes (*gB*1–4) [9]. A fifth *gB* genotype (*gB*5) was identified in many HIV patients [11]. Furthermore, the monitoring of CMV infection is facilitated by *gB* antigen detection [10]. Despite advances in viral diagnostic tools in the world, still, there is a difficulty in the diagnosis of CMV virus among transplants in Sudan because it depends on serological tests which has a limited diagnostic value, due to immunosuppressive therapy that is causing delayed seroconversion of IgM. The IgM level can remain undetectable (there is a time lag between primary infection and IgM antibody production) [12]. To our knowledge, there is no published data about CMV genotyping. So, this study focused on detecting CMV DNA by real-time PCR and the circulating *gB* genotypes of CMV among Sudanese renal transplant recipients.

## 2. Methods

### 2.1. Study Design, Duration, and Population

This study was a descriptive cross-sectional study conducted in the Kidney Transplanted Association Hospital and Ahmed Gassim Hospital in Khartoum state in June 2014 to June 2016. Renal transplant recipients, who agreed to participate in this study, all age, of both gender with or without signs and symptoms of CMV infection, were included in this study.

### 2.2. Sampling Technique

This study is based on the nonprobability convenience sampling technique.

### 2.3. Ethical Consideration

The study proposal was approved by the ethical board of Sudan University of Science and Technology and approved by two hospital administrations. Informed consent was taken from each renal transplant recipient before enrolment into the study. Data and samples were collected after informing and agreement of renal transplant recipients about the purposes and importance of the study.

### 2.4. Specimen Collection

One hundred and four (*n* = 104) renal transplant recipients were selected for this study. Five ml of blood specimen was collected in an EDTA container from each individual. Plasma was separated and stored at −20 C° until being analyzed.

### 2.5. Quantitative Real-Time PCR for Detection and Viral Load Estimation

DNA was extracted from peripheral blood plasma according to the instruction of a QIAamp DNA mini kit (QIAGEN, Germany). CMV amplification and quantification (estimation of viral load) were done using hot-start quantitative real-time PCR kits (CMV Real-RT Quant kits (Sacace, Italy). Intraassay variability was included using duplicates of a CMV calibrator standard containing 10^2^ and 10^4^ to ensure CMV viral load accuracy and reproducibility in clinical specimens.

### 2.6. Glycoprotein B Genotyping

Genotyping of HCMV *gB* was carried out by nested PCR and sequencing of a highly diverse region of glycoprotein B.

### 2.7. Nested PCR for gB

Nested PCR glycoprotein B genotyping was performed using outer primer pairs *gB*1, (5′CAAGARGTGAACATGTCCGA3′), *gB*2 (5′GTCACGCAGCTGGCCAG3′) *gB*3, and inner primer pairs (5′TGGAACTGGAACGTTTGGC3′), and *gB*4 (5′GAAACGCGCGGCAATCGG3′) (Macrogen, Korea) [13]. For outer-nested PCR, the PCR mixture with a total reaction volume of 25 *μ*l, containing 1 *μ*l of both forward and reverse primers and 8 *μ*l DNA, was subjected for amplification to an initial denaturation step at 95°C for 10 minutes. DNA was amplified for 35 cycles as follows: denaturation at 95°C for 1 minute, primer annealing at 55°C for 1 minute, and followed by a step of elongation at 72 for °C 1 minute; the final elongation was at 72°C for 7 minutes. For inner-nested PCR, the PCR mixture with a total reaction volume of 25 *μ*l, containing 1 *μ*l of both forward and reverse primers and 1 *μ*l DNA product, was subjected for amplification to an initial denaturation step at 95°C for 10 minutes. DNA was amplified for 35 cycles as follows: denaturation at 95°C for 30 seconds, primer annealing at 54°C for 45 seconds, and followed by a step of elongation at 72°C for 30 seconds; the final elongation was at 72°C for 7 minutes [13]. The PCR products (520 bp) for outer-nested (305 bp) and for inner-nested PCR were subjected to gel electrophoresis on 1.5% agarose. Gel results were photographed using a gel documentation system.

### 2.8. DNA Sequencing

Sequencing was carried out from the inner (305 bp) PCR product. The DNA sequencing was performed for the 10 PCR product of the CMV *gB* gene. DNA purification and standard sequencing were performed for both strands of *gB* genes by Macrogen Company (Seoul, Korea).

### 2.9. DNA Sequence Similarity and Alignment

Nucleotide sequences of both merged strand *gB* CMV genes were searched for similarity BLASTn [14] (http://http://blast.ncbi.nlm.nih.gov/Blast.cgi). Then, multiple-sequence alignment of nucleotides and translated proteins was done by BioEdit software [15].

### 2.10. Mutant Sequence Analysis

The mutant nucleotides were confirmed by their reverse strands. I-mutant version 3 [16] was used to study the stability of the mutant protein. Chimera software version 1.9 was used to predict the tertiary model of protein [17].

### 2.11. Phylogenetic Tree

The phylogenetic tree of CMV *gB* genes and their evolutionary relationship with well-characterized reference strains obtained from the NCBI database was constructed by the neighbour-joining method with the bootstrap test of phylogeny in the Molecular Evolutionary Genetics Analysis (MEGA) program, version 6 [18]. Bootstrap resembling strategy and reconstruction was carried out 1000 times to confirm the reliability of the phylogenetic tree.

### 2.12. Data Analysis

Data were analyzed using the statistical package for social science software (SPSS v.11.5). A *p* value of <0.05 was considered significant for all statistical tests in the present study.

## 3. Results

**Figure 1:**
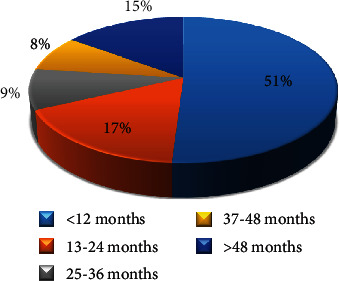
Postrenal transplantation time per month among the study group.

One hundred and four (*n* = 104) renal transplant recipients participated in this study; their age ranged from 11 to 72 years with a mean age of 37 ± 14.37 years (SD). Male recipients were 72 (69.2%), while 32 (30.8%) were females. Fifty (48%) of renal transplant recipients had received their organs in localized hospitals, while 54 (52%) received their organs abroad. Most of the renal transplant recipients received organs from 79 relative donors (76%) and only 25 (24%) from nonrelative donors. The mean total white blood cell count among renal transplant recipients was 7100 WBCs/cmm ± 2586.669 (SD) with a minimum count of 3200 WBCs/cmm and maximum count of 18600 TWBCs/cmm. The mean posttransplantation time in renal transplant recipients was 54 months, ranging from < one to 204 months. Less than one to 12 months represents 53 (51%), 13 to 24 months as 18 (17.3%), 25 to 36 months as 9 (8.8%), 37 to 48 months as 8 (7.7%), and >48 months as 16 (15.4%) of recipients (Figure 1).

**Figure 2:**
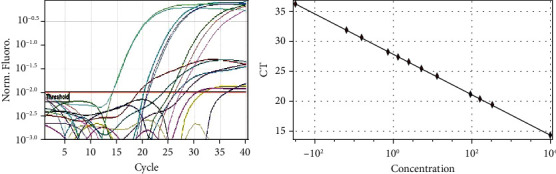
The standard curve for CMV (tested DNA). *r* = 0.99999. *r*ˆ2 = 0.99999. Efficiency = 1.00.

**Figure 3:**
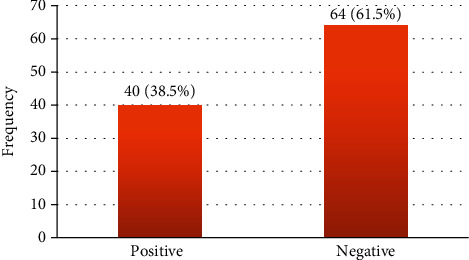
Frequency of positive CMV (viremia) among renal transplant recipients by qRT-PCR.

All plasma specimens (*n* = 104) were investigated for the presence of CMV DNA and viral load. Based on the constructed standard curve, the correlation coefficient was between 0.995 and 0.999, the amplification efficiency varied between 97% and 100%, and coefficient of variation (CV %) was from 0.00% to 8.5% for tested DNA and internal control for all trails (Figure 2). CMV DNA (viremia) was detected in 40/104 (38.5%) of renal transplant recipients (Figure 3). The average of the CMV DNA viral load was 358 × 10^4^ copies/ml (6.5 log_10_) ranging from 62 copies/ml (1.8 log_10_) to viral load 1.43 × 10^8^ copies/ml (9 log_10_). CMV viremia was detected in (60%), (17%), (10%), (5%), and (8%) of the recipients in <1–12, 13–24, 25–36, 37–48, and more than 48 months posttransplantation, respectively, and there was no significant difference (*p* value = 0.296) between CMV viremia and postrenal transplantation time.

Recipients with positive CMV viremia showed that CMV symptoms were 17/104 (16.3%), while 23/104 (22.1%) were positive CMV viremia and asymptomatic. The majority of CMV recipients with symptomatic infection had high viral loads (>1000 copies/ml) 14/17 (82.4%), and 3 (17.6%) had low viral loads (<1000 copies/ml). Of the 23 asymptomatic recipients, 1/23 (4.3%) had high viral loads, whereas 22/23 (95.7%) had low viral loads. The results revealed that the correlation between CMV loads of >1000 copies/ml and the presence of symptoms of CMV disease was highly significant (*p* ≤ 0.001). The medium CMV DNA viral load among symptomatic patients was 8.4 × 10^6^ copies/ml = 6.9 log_10_), and that in asymptomatic patients was 316 copies/ml = 2.5 log_10_). Individual DNA values for asymptomatic patients ranged between 62 and 1016 copies/ml (1.8 to 3 log_10_), whereas for symptomatic patients, they ranged from 537 to 1.43 × 10^8^ copies/ml (2.7 to 9 log_10_). The findings of this study indicated that fever 7 (41%), fever and leucopenia 6 (35%), and gastrointestinal disease 4 (24%) were the most common presenting symptoms of CMV disease.

**Figure 4:**
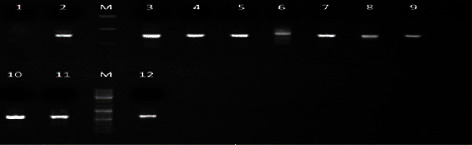
Gel electrophoresis of CMV UL55 gene PCR product (305 bp). M: marker (50 bp). Lane 1: negative control. Lanes 2, 3, 4, 5, 6, 7, 8, 9, 10, 11, and 12 are positive samples.

**Figure 5:**
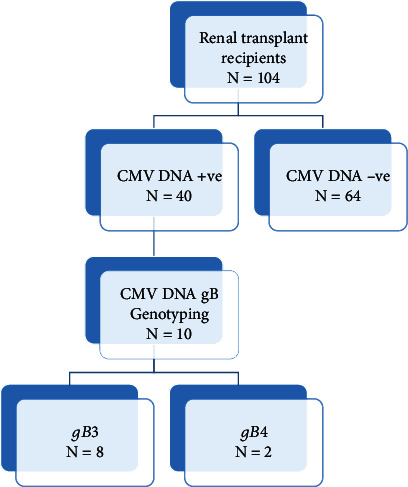
Flow chart illustrating the number of specimens investigated until genotyping.

**Figure 6:**
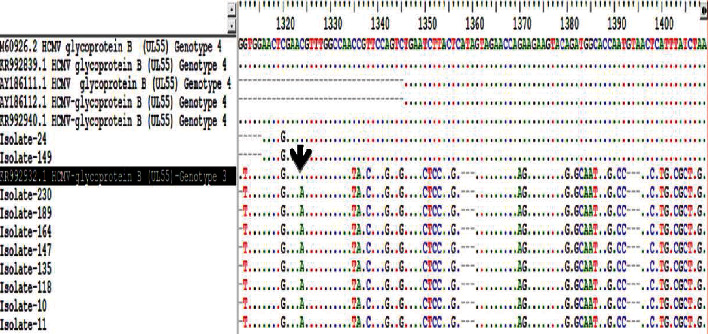
BioEdit multiple sequence alignment of the CMV gB gene compared to CMV reference strain from GenBank, where the black arrow indicates the transversion mutations in 8 CMV isolates.

Successful sequencing of CMV encoding *gB* was determined for ten samples of symptomatic Sudanese renal transplant recipients after performing nested PCR, with a *gB* gene (UL55) product of 305 bp (Figures 4 and 5). The nucleotide sequences of 10 isolates and their accession numbers were deposited in the GenBank database. The result of CMV genotyping by sequencing based on MEGA software revealed 8 cases (80%) for *gB*3 and 2 cases (20%) for *gB*4 genotypes among Sudanese renal transplant recipients. The most frequent genotype in HCMV-positive Sudanese renal transplant recipients was *gB*3, and no mixed genotypes were observed. BLAST nucleotide search showed that two isolates showed 99% identity with CMV *gB* genotype 4 (GenBank accession number M60926.2) from the United States of America, Spain (KR992839.1, KR992940.1), and Brazil (AY186111.1, AY186112.1). Eight isolates showed 100% identity with CMV *gB* genotype 3 (KR992932.1) from Spain (Figure 6).

**Figure 7:**
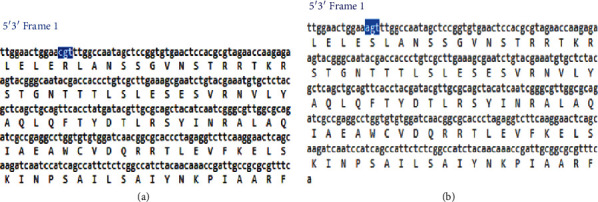
(a) Normal codon and protein sequence of CMV *gB* wild type from GenBank versus (b) mutant codon and protein as indicated by the blue colour.

**Figure 8:**
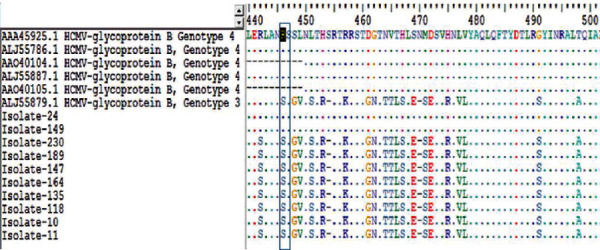
Amino acid multiple sequence alignment of Sudanese mutant gB gene compared to other gB genes from the database. Substitution of the amino acid arginine (R) to serine (S) as indicated by the black colour.

**Figure 9:**
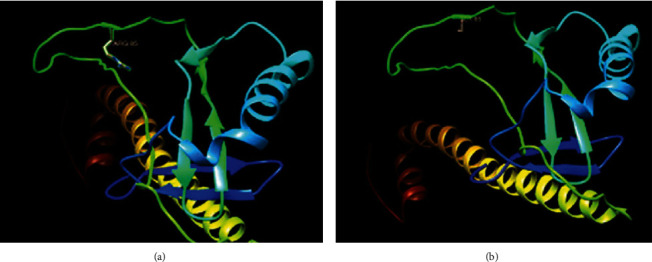
Tertiary protein structure of wild (a) and mutant (b) *gB*3 genes of isolates. The predicted amino acid arginine at position 85 from GenBank predicted by Chimera software version 1.9.

**Figure 10:**
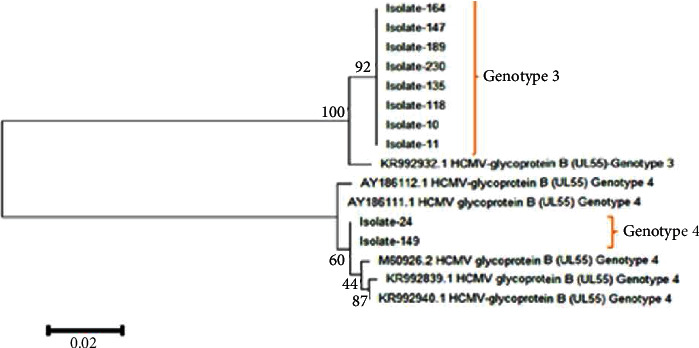
Phylogenetic tree based on *gB* gene sequences of 10 CMV isolates from renal transplant recipients. The phylogenetic tree analysis was constructed using the neighbour-joining method in MEGA.

Multiple-sequence alignment of obtained CMV *gB* sequences compared with reference sequences previously published in the database exhibited transversion mutation in 8 isolates in which A replaced C at position 253 from reference CMV *gB* 3 (KR992932.1) (Figure 7). That resulted in a substitution of the codon CGT arginine (R) to AGT serine (S) (Figure 8). Substitution of the protein was shown by the tertiary protein structure of the wild type (R) and mutant type (S) at position 85 (Figure 9). This substitution resulted in a decrease in protein stability as indicated by I-mutant software. The phylogenetic tree analysis was performed to compare the genetic distances and the evolutionary lineage for all ten isolates with well-characterized reference isolates from GenBank, (Figure 10).

## 4. Discussion

Cytomegalovirus infection is one of the most frequently encountered opportunistic viral pathogens in renal transplantation [19]. This study was designed to determine the frequency of CMV infection and its *gB* genotype distribution among Sudanese renal transplant recipients. The study population was 104 renal transplant recipients. The male/female ratio is about 2 : 1. This finding is in agreement with Khameneh et al. [20] in Iran, males were 61.1%, and females were 38.9%; Also, Hasanzamani et al. [19] reported that in Iran, 41(62.1%) of the population were male, while 25(37.9%) were female. Most recipients in the present study received triple immunosuppressive therapy that makes them more liable to CMV infection as reported by Nafar and his colleagues [21] which indicate that high immunosuppressive regimen is associated with a higher risk for CMV infection. Al-Alousy et al. [22] observed that the type, intensity of immunosuppressive therapy, and the level of immunosuppression act as a critical exogenous factor influencing the HCMV reactivation following transplantation such as cyclosporine.

The current study showed that CMV DNA (viremia) was detected in 38.5% of renal transplant recipients using quantitative real-time PCR. These results are relatively higher than those observed by Tong et al. [23] (22%), Madi et al. [24] in Kuwait (24%), Enan et al. [25] in Sudan (32.7%), and Lashini et al. [26] in Iran (25.9%). Similar findings to our results were observed by Garrigue et al. [27] (36.6%) and Zhang et al. [28] in China (37.7%). In contrast, the lower result was obtained by Cordero et al. [29] in the Philippines (5.8%), Cupic et al. [30] in Serbia (12.5%), and Khalafkhany et al. [31] in Iran (15.9%). No antiviral prophylactic or preemptive therapy may explain the higher frequency of CMV among this study group.

It is of interest to observe that the average of CMV DNA viral load was 358 × 10^4^ copies/ml (6.5 log_10)_ ranging from 62 copies/ml (1.8 log_10_) to 1.43 × 10^8^ copies/ml (8.2 log_10_). The lack of screening in most patients probably explains the high viral loads at diagnosis and the large variation in viral loads.

In the present study, 51% of the population had posttransplantation time from less than one to 12 months. This finding increases the possibility of primary CMV infection or reactivation. The frequency of CMV viremia from the total positive was higher in the first 12 months of transplantation 24/40 (60%) compared with the later onset. Similar results were observed by Khalafkhany and his colleagues [31] in Iran who detected CMV viremia in 31.2% of 0–3 months, 30.7% of 4–6 months, and 17.5% of 7–12 months posttransplantation.

In this study, higher viral load correlates precisely with the development of CMV-related symptoms and viral loads were slightly lower with asymptomatic patients (high significant difference, *p* ≤ 0.001), in which 82.4% of patients had clinical symptoms of CMV disease with viral loads > 1000 copies/ml. These observations confirm previous reports by Hadaya and her colleagues [32], Knipe and Howley [33], Madi et al. [24], Helanter et al. [34], and Rangbar-Kermani et al. [35]. A medium level of viral load was higher in patients with symptomatic CMV disease than asymptomatic disease. The discrepancies are in three symptomatic patients (17.6%) with a viral load of <1000 copies/ml and one asymptomatic patient (4.3%) with a viral load of >1000 copies/ml. These discrepancies could be explained by several factors such as the source of the donor's kidney, nature of immunosuppressive disease, and genotypes of the virus.

Findings of this study indicated that fever, fever leucopenia, and gastrointestinal disease with abdominal pain and diarrhoea were the most common presenting symptoms of CMV disease. Similar results were obtained by Ardalan [36] who reported that most symptomatic CMV infections manifest as fever, fatigue, and cytopenia and the gastrointestinal tract is the most common site of tissue-invasive CMV infection.

The result of sequencing and genotyping of the HCMV *gB* gene (UL 55) for 10 CMV isolates revealed that *gB*3 (80%) was the most frequent genotype among Sudanese renal transplant recipients, whereas gB4 was 20% and no mixed genotypes were observed. No published data is available in Sudan on CMV *gB* genotyping neither in renal transplant recipients nor the immunocompetent host with CMV infection. These results are in agreement with previous reports in Italy by Arista et al. [37] in which the predominant circulation of HCMV strains was *gB* types 2 and 3. Somewhat similar results were reported by De Vries et al. [38], in Netherland; *gB*1 and *gB*3 were the most common genotypes in kidney transplant recipients and congenitally infected newborns. Gandhoke et al. [12] in India found that *gB*3 was the most prevalent genotype in symptomatic infants. The results of this study differ from previous studies undertaken in other parts of the world. Pacsa et al. [39] in Kuwait reported that *gB*1 (27%) was the most frequent genotype followed by *gB*2 (25%), *gB*3 (19%), and *gB*4 (1%) and mixed genotypes were 27%. Coaquette et al. [40] in France indicated that *gB*1 was found in 28.9% of patients, *gB*2 (19.6%), *gB*3 (23.7%), *gB*4 (2.0%), and mixed infection (25.8%). Dieamant et al. [41], found that *gB*1 and *gB*2 were the most common genotypes in Brazilian pediatric kidney transplant patients. Khalafkhany et al. [31] in Iran mentioned that *gB*1 (26.5%), *gB*2 (20.5%), *gB*3 (17.6%), and *gB*4 (5.9%) genotypes were detected. Mixed genotype infection was observed in 29.4% of the recipients. The substantial differences in genotype frequencies in this study compared to previous studies might, in part, be due to variation in the geographical distribution of the CMV genotypes. In the current study, no mixed genotypes were observed and this might be due to the low number of the individual clone being sequenced down to the level of 5%. Besides, mixed infections accounted for roughly one quarter to one-half of HCMV infections over a wide range of human populations, as mentioned by Renzette et al. [42].

The results of genotyping and sequencing in this study represent the first genetic characterization of HCMV in Sudan. Transversion mutations in the *gB* ggene identified in eight of Sudan's *gB*3 genotypes result in amino acid substitutions and reduce protein stability. The obtained results of the protein tertiary structure showed a difference in size between the wild type, which has larger residue, and the mutant type. This difference is probably altering or particularly increasing viral pathogenicity, as the *gB* gene is one of the essential envelope glycoproteins of HCMV, implicated in virus entry, cell-to-cell spread, and the fusion of infected cells [10]. The variability and mutations, particularly in *gB* that arise, can be advantageous to the virus, increasing viral fitness and adaptation [43]. Findings of phylogenetic analysis in this study indicated that the HCMV was related to several strains worldwide that are far from Sudan (e.g., USA, Spain, and Brazil). It is believed that their presence reflects the broader circulation of these strains in our geographical area and worldwide for both renal transplant recipients as well as immunocompetent with primary HCMV infection or disease. In this study, only ten isolates were subjected to sequencing due to financial constraints.

## 5. Conclusions

This study concluded high frequency of CMV infection among Sudanese renal transplant recipients. CMV viral loads were slightly lower in asymptomatic patients. In this study, CMV *gB*3 is considered the most predominant glycoprotein B genotype in Sudanese renal transplant recipients.

We recommended that early monitoring of CMV using a sensitive method such as qRT-PCR that precisely detects viral replication and provides guiding information will help to initiate preemptive antiviral therapy that might have the advantages of reducing the occurrence of CMV disease.

## Data Availability

The nucleotide sequences were submitted to GenBank with the following accession numbers: MF179785, MF179786, MF179787, MF179788, MF179789, MF179790, MF179791, MF179792, MF179793, and MF179794.

## Abbreviations

CMV: Cytomegalovirus
DNA: Deoxyribonucleic acid
gB: Glycoprotein B
qRT-PCR: Quantitative real-time PCR.
